# Lipocalin 2 – mutation screen and serum levels in patients with anorexia nervosa or obesity and in lean individuals

**DOI:** 10.3389/fendo.2023.1137308

**Published:** 2023-03-21

**Authors:** Yiran Zheng, Luisa Sophie Rajcsanyi, Manuela Kowalczyk, Johanna Giuranna, Beate Herpertz-Dahlmann, Jochen Seitz, Martina de Zwaan, Wolfgang Herzog, Stefan Ehrlich, Stephan Zipfel, Katrin Giel, Karin Egberts, Roland Burghardt, Manuel Föcker, Saad Al-Lahham, Johannes Hebebrand, Dagmar Fuhrer, Susanne Tan, Denise Zwanziger, Triinu Peters, Anke Hinney

**Affiliations:** ^1^ Department of Child and Adolescent Psychiatry, Psychosomatics and Psychotherapy, University Hospital Essen, University of Duisburg-Essen, Essen, Germany; ^2^ Center for Translational Neuro- and Behavioral Sciences, University Hospital Essen, University of Duisburg-Essen, Essen, Germany; ^3^ Department of Endocrinology, Diabetes and Metabolism and Clinical Chemistry – Division of Laboratory Research, University Hospital Essen, University of Duisburg-Essen, Essen, Germany; ^4^ Department of Child and Adolescent Psychiatry and Psychotherapy, University Hospital of the RWTH Aachen, Aachen, Germany; ^5^ Department of Psychosomatic Medicine and Psychotherapy, Hannover Medical School, Hannover, Germany; ^6^ Department of Internal Medicine II, General Internal and Psychosomatic Medicine, University of Heidelberg, Heidelberg, Germany; ^7^ Translational Developmental Neuroscience Section, Department of Child and Adolescent Psychiatry, TU-Dresden, University Hospital Carl Gustav Carus, Dresden University of Technology, Dresden, Germany; ^8^ Eating Disorders Research and Treatment Center, Department of Child and Adolescent Psychiatry, Faculty of Medicine, TU Dresden, Dresden, Germany; ^9^ Department of Psychosomatic Medicine and Psychotherapy, Medical University Hospital, Tübingen, Germany; ^10^ Centre of Excellence for Eating Disorders, University of Tübingen, Tübingen, Germany; ^11^ Department of Child and Adolescent Psychiatry, Psychosomatics and Psychotherapy, University of Würzburg, Würzburg, Germany; ^12^ Department of Child and Adolescent Psychiatry, Psychosomatics and Psychotherapy, Oberberg Fachklinik Fasanenkiez, Berlin, Germany; ^13^ Department of Child and Adolescent Psychiatry, University of Münster, Münster, Germany; ^14^ Department of Biomedical Sciences, Faculty of Medicine and Health Sciences, An-Najah National University, Nablus, Palestine; ^15^ Department of Endocrinology, Diabetes and Metabolism, University Hospital Essen, University of Duisburg-Essen, Essen, Germany

**Keywords:** Energy homeostasis, bone marrow, secondary structure of protein, GWAS, lean body mass (LBM)

## Abstract

**Context:**

The bone-derived adipokine lipocalin-2 is relevant for body weight regulation by stimulating the leptin-melanocortin pathway.

**Objective:**

We aimed to (i) detect variants in the lipocalin-2 gene (*LCN2*) which are relevant for body weight regulation and/or anorexia nervosa (AN); (ii) describe and characterize the impact of *LCN2* and *MC4R* variants on circulating lipocalin-2 level.

**Methods:**

Sanger sequencing of the coding region of *LCN2* in 284 children and adolescents with severe obesity or 287 patients with anorexia nervosa. *In-silico* analyses to evaluate functional implications of detected *LCN2* variants. TaqMan assays for rare non-synonymous variants (NSVs) in additional independent study groups. Serum levels of lipocalin-2 were measured by ELISA in 35 females with NSVs in either *LCN2* or *MC4R*, and 33 matched controls without NSVs in the two genes.

**Results:**

Fourteen *LCN2*-variants (five NSVs) were detected. *LCN2*-p.Leu6Pro and p.Gly9Val located in the highly conserved signal peptide region may induce functional consequences. The secondary structure change of lipocalin-2 due to *LCN*2-p.Val89Ile may decrease solubility and results in a low lipocalin-2 level in a heterozygotes carrier (female recovered from AN). Lean individuals had lower lipocalin-2 levels compared to patients with obesity (*p* = 0.033).

**Conclusion:**

Lipocalin-2 levels are positively associated with body mass index (BMI). Single *LCN2*-variants might have a profound effect on lipocalin-2 levels.

## 1 Introduction

Body weight regulation is based on energy intake and expenditure. When energy balance is disturbed, a number of disorders can ensue, such as obesity (1) and anorexia nervosa (AN). Obesity is a global health hazard and is in adults commonly defined with a body mass index (BMI, kg/m^2^) at or above 30 kg/m^2^ (2–4), and with BMI at or above the 97^th^ percentile (5) in children and adolescents. AN is a life-threatening disorder accompanied with severely low body weight (6) (DSM-IV (7) and DSM-5 criteria (8). Genetic and environmental factors influence energy homeostasis (9).

Lipocalin-2 was initially considered as an adipokine highly expressed by murine fat cells (10) and later recognized as a bone-derived hormone associated with appetite regulation (11). A study in mice demonstrated that lipocalin-2 could reduce appetite and decrease fat mass *via* crossing the blood-brain barrier and binding to the melanocortin 4 receptor protein (MC4R) in the hypothalamus (11). Mosialou et al. showed that MC4R is necessary for lipocalin-2 to regulate appetite in a *Mc4r* knockout mouse model (11).

The MC4R plays an essential role in the leptin-melanocortin pathway and thus in energy homeostasis (12). Mutations in the MC4R gene (*MC4R*) display the most common cause of monogenic obesity (13) and affect 2-4% of severely obese individuals (14). Patients with AN show increased α-MSH-reactive IgG, leading to rapid MC4R internalization and potentially improved satiety and reduced hunger (15). The first *Mc4r* knockout mouse model was generated in 1997. A dominant effect of *Mc4r* mutations of body weight was reported, whereby the effect was more pronounced in female mice (16). In humans, different mutations in *MC4R* have a major gene effect in obesity (17–20). Up to now more than 160 different mutations in *MC4R* had been reported mainly in severely obese individuals (12).

We had shown that *MC4R* and lipocalin-2 gene (*LCN2*) mutations were detected in 2.42% and 0.84%, respectively, of Spanish children with obesity. Some individuals with functionally relevant mutations in *MC4R* or *LCN2* were able to reduce their BMI-SDS in a lifestyle intervention (21). We hypothesized that mutations in *MC4R* or *LCN2* may have an effect on the lipocalin-2 level and thus influence weight regulation.

Bone marrow, where lipocalin-2 is mainly expressed, consists of red (hematopoietic) and yellow (adipose tissue) components. Although lipocalin-2 can be secreted by both hematopoietic and bone marrow adipose tissue (BMA) cell types, the expression level is significantly higher in osteoblasts (22). Therefore, the expression level of lipocalin-2 may be affected by the phase of accelerated marrow adiposity accumulation and the total bone mass. BMI is a proxy indicator for body weight without describing the composition. Body weight is composed of body fat (BF) and lean body mass (LBM). LBM has been declared to be positively associated with measurements of bone mass or density for decades (23, 24). Thus, we expect a correlation between lipocalin-2 level and BMI or LBM.

## 2 Methods

### 2.1 Study population

#### 2.1.1 Mutation screen and TaqMan assay

To detect variants in *LCN2*, 571 German individuals consisting of (i) 287 females with diagnosed AN [acute or recovered, diagnosed with DSM-IV criteria (7)], (ii) 284 children, adolescents and young adults (younger than 25 years of age (25) with (severe) obesity [91.7% individuals were severely obese with BMI percentage ≥ 97^th^ percentile (5)] were Sanger sequenced. TaqMan assays for detected rare non-synonymous variants (NSVs) were performed in additional independent study groups consisting of (i) 170 German females with AN, (ii) 200 children or adolescents with severe obesity (99.5% individuals younger than 25 years of age).

#### 2.1.2 LBM calculation for all analyzed individuals

LBM was calculated for each individual with the equations shown in the **Supplementary Material (Method 1)**. The LBM percentage (LBM%) refers to the percentage of LBM within the total body weight.

#### 2.1.3 ELISA assay

For the analysis of lipocalin-2 level in female individuals of whom serum was available, we used (i) two heterozygous carriers of NSVs in *LCN2* (n = 2), (ii) individuals heterozygous for 12 *MC4R* NSVs (n = 33) and (iii) matched (for disorder, sex, age (± 3 years), LBM% (± 3%)) controls homozygous for a reference genotype at the respective genes (n = 33).

*MC4R* NSVs were derived from our previous studies (Rajcsanyi et al. unpublished data (14, 19, 26, 27), which contained mutation screens for a total of 4,985 individuals of German descent. All individuals involved in the *LCN2* mutation screen and ELISA assay were sequenced for *MC4R* variants in our previous studies. Additional Sanger sequencing for *LCN2* was performed for the individuals for whom *LCN2* genotypes were not available: (i) female patients with AN (n = 7), (ii) children or adolescents with obesity (n = 39), and (iii) healthy-lean individuals (BMI percentage ≤ 15^th^ percentile, n = 9).

Briefly, the 464 unrelated female patients with AN included 381 individuals with acute AN and 83 individuals with AN history were recruited in our study. The patients with acute AN had a mean age of 19.47 (SD = 7.68) years and a mean BMI of 15.6 (SD = 1.8) kg/m^2^. The individuals recovered from AN had a mean age of 27.09 (SD = 9.88) years and a mean BMI of 20.56 (SD = 2.75) kg/m^2^. The phenotype distribution of analyzed study groups is shown in **Table 1**. All participants gave written informed consent in case of minors their parents. The study was approved by the Ethics Committees of the Universities of Aachen, Dresden, Essen, Frankfurt, Hannover, Heidelberg, Marburg, Tübingen and Würzburg, and was performed in accordance with the Declaration of Helsinki.

**Table 1:** Phenotypes of the study groups.

Diagnosis	Sex	Statistic	Age	BMI (kg/m^2^)	LBM (kg)^1^	LBM%^2^
AN	100% Female	Mean (SD)^3^	20.8 (8.6)	16.48 (2.76)	38.23 (5.65)	85.14 (5.09)
	464 (294)^4^	[Min, Max]^5^	[11.7, 67.4]	[9.03, 29.22]	[22.47, 57.16]	[63.51, 96.2]
OB	All	Mean (SD)	14.3 (3.7)	33.28 (6.88)	54.8 (12.18)	63.86 (8.45)
	523 (323)	[Min, Max]	[3.4, 39.2]	[20.3, 63.42]	[18.43, 93.4]	[33.46, 79.12]
	59.7% Female	Mean (SD)	14.4 (3.7)	33.39 (7.24)	52.56 (9.7)	61.98 (9.48)
	312 (190)	[Min, Max]	[5.6, 24.4]	[20.35, 63.42]	[20.41, 77.12]	[33.46, 79.12]
	40.3% Male	Mean (SD)	14.2 (3.8)	33.13 (6.32)	93.4 (13.82)	77.32 (5.6)
	211 (133)	[Min, Max]	[3.4, 39.2]	[21.88, 56.86]	[18.43, 93.4]	[47.85, 77.32]
Lean	100% Female	Mean (SD)	21.7 (1.9)	17.2 (0.61)	42.96 (1.32)	84.17 (1.53)
	9 (9)	[Min, Max]	[19.6, 24.1]	[16.16, 18.17]	[40.88, 44.39]	[81.68, 86.82]

^1^LBM: Lean body mass in kg; ^2^LBM%: the percentage of LBM in the total body weight; ^3^Mean (SD): average value (standard deviation); ^4^j(k): total samples used for TaqMan analyses and Sanger sequencing; in brackets: Sanger sequenced samples; ^5^[Min, Max]: the interval of value from minimum to maximum.

### 2.2 Mutation screen

The transcript variant 1 (LCN2-201, ENST00000277480.7) of *LCN2* (Chr9: 128,149,071 ~ 128,153,453, GRCh38.p13) was extracted from the Ensembl Database (28) (http://www.ensembl.org/index.html). Its coding region was divided into four PCR fragments [(primers located in the introns, **Supplementary Material Method 2**)]. The polymerase chain reaction (PCR) amplified DNA samples were sequenced unidirectional by Microsynth Seqlab GmbH (Göttingen, Germany). All sequenced samples passed internal quality control and were genotyped with SeqMan Pro software (v.11.0.0, DNAstar, Inc., Madison, WI, USA) by two experienced individuals independently. Samples with variant pattern were confirmed with sequencing of the other strand. Hardy-Weinberg Equilibrium (HWE) was performed for all analyzed variants.

### 2.3 *In-silico* functional analyses on detected variants in *LCN2* and *MC4R*

#### 2.3.1 GWAS look-up for detected variants in GWASs and linkage disequilibrium analysis

Detected variants with dbSNP numbers were looked up in the GWAS for BMI by Pulit et al. (for females, males and combined sexes) (29) and in the GWAS for AN by Watson et al. (30). The data of variants located in the genomic region upstream and downstream (±) 500kb of *LCN2* was extracted from GWAS summary statistics (**Supplementary Material Tables 1, 2**) and plotted in GraphPad Prism 9.4.0. The detected *LCN2* variants were analyzed for LD scores with genome-wide significant variants in GWAS for BMI (29) which were located in the genomic ± 500kb region in LD matrix (31) (https://ldlink.nci.nih.gov/?tab=ldmatrix, population: European, genotype data from 1000G Project). If the LD scores between detected variants and GWAS hits were D’ > 0.6 and R^2^ > 0.3, the paired variants were analyzed for haplotype in LDpair (31) (https://ldlink.nci.nih.gov/?tab=ldpair).

#### 2.3.2 Conservation analysis on detected variants

The conservation analysis for detected single nucleotide *LCN2* variants was performed by human *LCN2* gDNA and 30 different species from three superorders (ten primates, ten rodents and related species, ten laurasiatherian, **Supplementary Material Table 3**). The gDNA sequences align was utilized the cluster W method in the software MegAlign by DNAStar, Inc. (version 10.1.0). The conservation percentiles (Cper.) were calculated for all detected variants. The variants with a value of Cper. larger than 85% were identified as “highly conserved”.

#### 2.3.3 Recruited *in-silico* tools analyses

All detected *LCN2* variants and the *MC4R* variants involved in ELISA analysis were analyzed for deleteriousness and mRNA splicing pattern alteration due to nucleotide exchange, protein stability and secondary structure variance caused by non-synonymous variants by 12 *in-silico* tools. The procedure of variants in-silico analyses can be found in **Supplementary Material Method 3**. We then looked up previous functional analyses for MC4R and classified the tested mutation in our study into GOF (gain of function), RF (reduce function), and LOF (loss of function).

### 2.4 ELISA assays for lipocalin-2 serum levels

The blood sampling of 35 females harboring *LCN2* or *MC4R* variants and 33 age and sex-matched controls without mutations in these genes was performed in the morning after an overnight fast. Serum samples were stored at -80°C and accurate temperature was controlled by an in-house master display. Six serum samples (two from *LCN2*-variant carriers, four from *MC4R*-variant carriers) were measured twice. For the repeated samples mean values were calculated. Circulating serum lipocalin-2 concentrations were measured using a quantitative sandwich enzyme immunoassay (Human Lipocalin-2/NGAL Quantikine ELISA Kit, Catalog: DLCN20, RRID: AB_2894833, R&D Systems, UK, Abingdon) and optical densities were detected using the SpectraMax M5 microplate reader (Molecular Devices Germany GmbH, Germany, Munich) according to the manufacture’s instructions. The intra-assay variation was < 4.4%, the inter-assay variation was < 7.9% and the detection limit was 0.012 ng/ml (according to the product insert). The 95% confidence intervals (CI) for matched controls were calculated in R studio (version: 2022.12.0 + 353 for MAC). ELISA results were plotted in R studio by the “lattice” package (32). The evaluated samples with phenotypes are shown in **Supplementary Material Table 4**.

### 2.5 TaqMan

The identified rare missense variants in *LCN2* (p.Gly9Val, rs147787222; p.Val89Ile, rs200876706; p.Arg174Ser, rs546790138) were genotyped in larger study groups by performing TaqMan assays (Thermo Fisher Scientific, Inc., Waltham, MA, USA). The phenotypes of the individuals used for the three mutated genotypes determinations are summarized in **Table 1**.

### 2.6 Statistics

Association analyses between the detected variants and phenotypes were performed with Fisher’s exact test (**Supplementary Material Method 4**). Associations between LBM%, BMI and lipocalin-2 levels were analyzed with non-parametric Spearman correlation analyses. The differences in measurements for lipocalin-2 levels between groups were tested with Mann-Whitney U or Kruskal-Wallis 2-way ANOVA. Analyses were performed using IBM ^®^ SPSS ^®^ Statistics v29.0.0 for Windows. Exact two-sided significances were calculated, the alpha level was set to 0.05. To control for the overall type I error rate, Bonferroni correction was applied.

### 2.7 Study procedure

**Figure 1:**
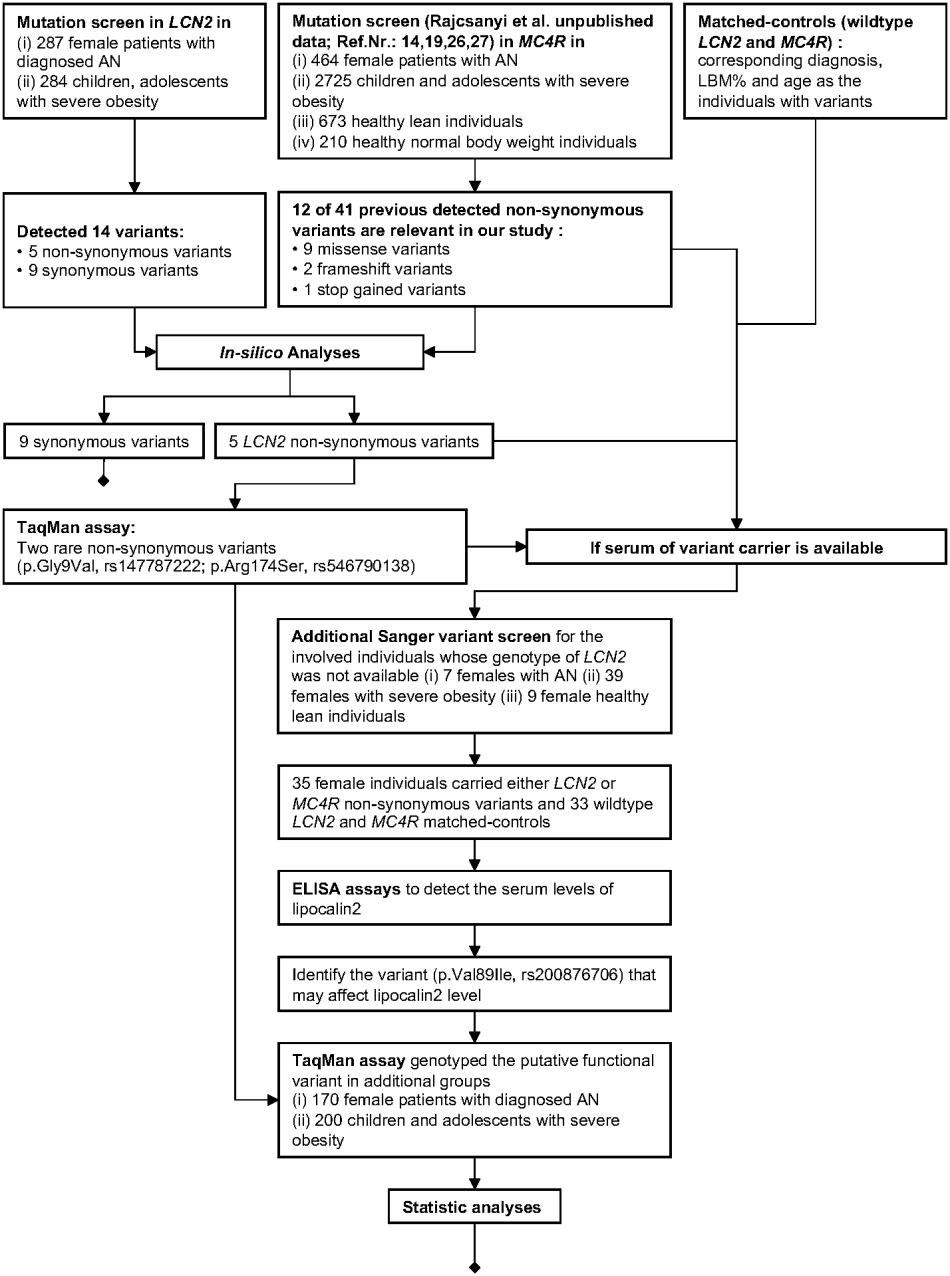
Workflow of our study.

Our study design is shown in the following workflow figure (**Figure 1**).

## 3 Results

### 3.1 Detected *LCN2* variants

**Figure 2:**
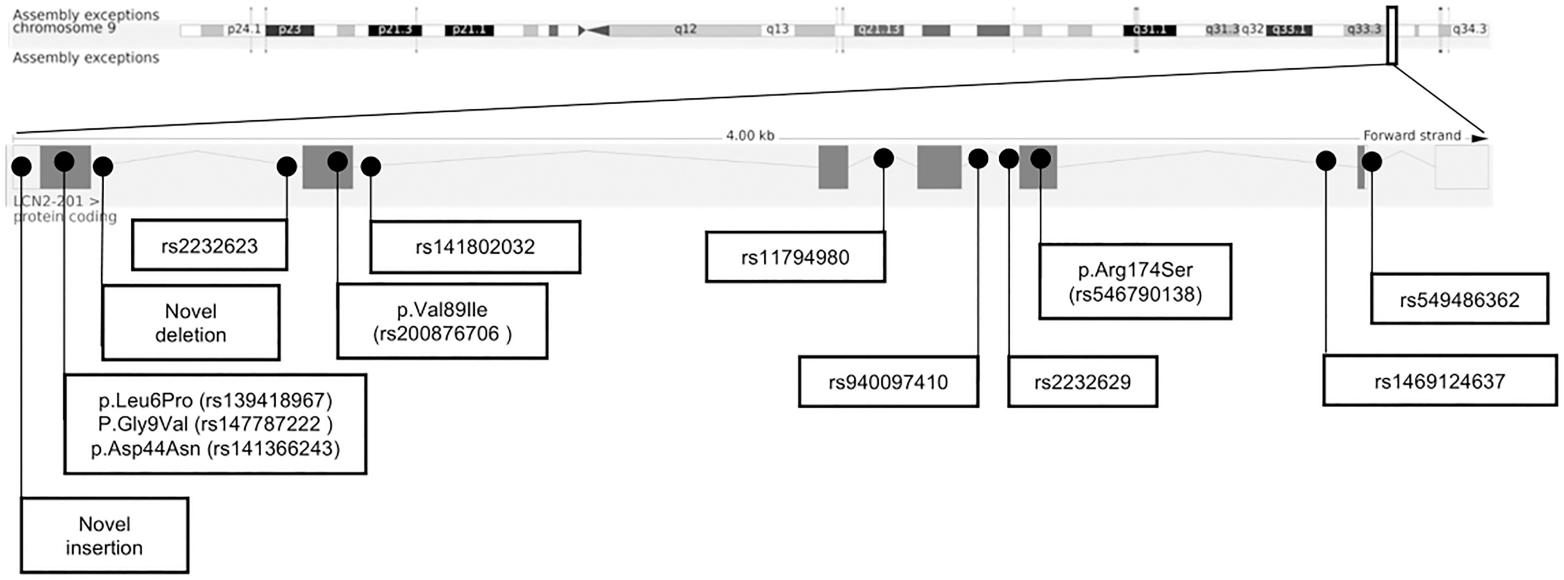
Chromosomal structure of *LCN2* (GRCh38.p13, Ensembl) and genomic location of 14 detected variants (28).

We initially Sanger-sequenced the *LCN2* gene in 287 female patients with AN and 284 children, adolescents with severe obesity. Seven patients with AN and 39 children or adolescents with obesity were then Sanger sequenced for ELISA assay. All six coding exons of *LCN2* and intronic parts flanking the coding regions were sequenced. Fourteen variants were detected, including five coding non-synonymous variants, six intronic SNPs, two intronic deletions and one novel intronic insertion (**Figure 2**). The genotype distribution of all detected variants is shown in **Supplementary Material Tables 5-1**).

Three missense variants (p.Leu6Pro, p.Asp44Asn, p.Val89Ile) were identified in both study groups. Two additional missense variants (p.Gly9Val, p.Arg174Ser) were only detected once in two female patients with obesity, each. For these two missense variants TaqMan assays were performed in additional independent study groups (170 females with AN; 200 children and adolescents with obesity). To sum up, 464 females with AN and 523 children or adolescents with severe obesity were genotyped for p.Gly9Val and p.Arg174Ser (**Supplementary Material Tables 5-2**). In total, two females with AN, two females and one male with obesity were heterozygous for p.Gly9Val. For p.Arg174Ser no additional variant carriers were identified.

The genotype frequencies of all detected variants were in the HWE. The genotype distribution of detected missense and structural variants are shown in **Table 2**.

**Table 2:** The genotype distribution of detected missense and structure variants in *LCN2 via* Sanger sequencing and TaqMan assays.

dbSNP_ID		Inser_GCGCCT^1^	rs139418967	rs147787222	rs141366243	Del_G^2^	rs200876706	rs546790138	rs549486362
Position (GRCh38.p13)		9:128149499-128149500	9:128149542	9:128149551	9:128149655	9:128149671	9:128150364	9:128152227	chr9:128153211-128153223
Location (*LCN2*)		Intron 1	Exon 2	Exon 2	Exon 2	Intron 2	Exon 3	Exon 6	Intron 7
Allele1/Allele2		-/GCGCCT	T/C	G/T	G/A	G/-	G/A	C/A	TG(C)_6_/-
AA exchange		NA.^3^	p.Leu6Pro	p.Gly9Val	p.Asp44Asn	NA.	p.Val89Ile	p.Arg174Ser	NA.
AN^4^	11	293	293	462	292	293	463	464	293
	12	1	1	2	2	1	1	0	1
	22	0	0	0	0	0	0	0	0
OB^5^	11 (f/m)^6^	323 (191/132)	322 (191/131)	520 (310/210)	321 (190/131)	323 (191/132)	522 (311/211)	522 (311/211)	322 (190/132)
	12 (f/m)	0	1 (0/1)	3 (2/1)	2 (1/1)	0	1 (1/0)	1 (1/0)	1
	22 (f/m)	0	0	0	0	0	0	0	0

^1^Inser_GCGCCT: detected novel intronic insertion; ^2^Del_G: detected novel intronic deletion; ^3^NA.: not applicable; ^4^AN: For the missense variants (p.Gly9Val, p.Val89Ile and p.Arg174Ser) the sample size is 464 (294 from Sanger sequencing and 170 from TaqMan assays); ^5^OB: For the non-synonymous variants (p.Gly9Val, p.Val89Ile and p.Arg174Ser) the sample size is 523 (323 from Sanger sequencing and 200 from TaqMan assays); ^6^f/m: the count of detected corresponding genotype in female/male.

### 3.2 *In-silico* analyses

#### 3.2.1 Analyses for *LCN2* variants

##### 3.2.1.1 Association analysis for detected *LCN2* variants and obesity or AN

All detected variants were in HWE. None of the detected variants was associated with AN nor obesity (**Supplementary Material Tables 6-1, 2**).

##### 3.2.1.2 Two missense *LCN2* variants with putative functional consequences

The *in-silico* analyses were applied in three dimensions deleteriousness analyses on nucleotide changes (seven predictors), putative splicing site alteration analyses (two predictors) and likelihood of protein stability reduction due to single amino acid changes (four predictors). When the detected variants were predicted as pathogenic in at least one predictor of all three dimensions and Cper. value higher than 85%, we assumed that the variant may have functional consequences. As shown in **Table 3** (detailed data shown in **Supplementary Materials Tables7–9**), the two missense variants (p.Leu6Pro and p.Gly9Val) are highly conserved with Cper. values above 90% and were predicted as pathogenic in all *in-silico* analyses dimensions.

**Table 3:** In-silico analyses for detected variants in *LCN2* and those *MC4R* variants subjected to ELISA assays.

Detected variants in *LCN2*						Analyzed variants in *MC4R*					
dbSNP ID	AA change^1^	Cper.^2^ (%)	Deleteriousness^3^	Splicing site^5^	Protein stability^7^	dbSNP ID	AA change	Deleteriousness	Splicing site	Protein stability	Functional analyses
			i/n^4^	j/k^6^	z/g^8^			i/n	j/k	z/g	Prediction
**rs139418967**	p.Leu6Pro	100	4/7	2/2^12^	2/2	**rs13447323**	p.Ser30Phe	3/6	0/2	1/2	RF^9^ (33)
**rs147787222**	p.Gly9Val	93.55	2/6	1/2	1/2	**rs13447324**	p.Try35stop	7/7	NA.	NA.	LOF^10^ (33, 34)
**rs141366243**	p.Asp44Asn	80.65	1/7	1/2	3/4	**rs121913557**	p.Val50Leu	5/6	2/2	3/4	RF (33)
**rs2232623**	NA.^13^	41.94	0/7	1/2	NA.	**rs2229616**	p.Val103Ile	5/6	1/2	3/4	GOF^11^ (35, 36)
**rs200876706**	p.Val89Ile	16.13	1/7	2/2	4/4	**rs13447329**	p.Thr112Met	2/6	2/2	2/2	RF (14, 37)
**rs141802032**	NA.	35.48	3/7	2/2	NA.	**rs13447330**	p.Ile121Thr	7/7	1/2	4/4	RF (19, 35)
**rs11794980**	NA.	61.29	1/7	1/2	NA.	**rs13447331**	p.Ser127Leu	7/7	2/2	2/4	LOF (38)
**rs940097410**	NA.	61.29	2/7	1/2	NA.	**rs13447332**	p.Arg165Trp	7/7	2/2	3/4	LOF (39, 40)
**rs374443333**	NA.	45.16	1/7	1/2	NA.	**rs121913563**	p.Ala175Thr	3/6	2/2	4/4	RF (34)
**rs2232629**	NA.	9.68	1/7	2/2	NA.	**rs13447338**	p.Leu211fsx	1/1	NA.	NA.	LOF (41)
**rs546790138**	p.Arg174Ser	77.42	0/7	1/2	4/4	**rs52820871**	p.Ile251Leu	3/7	2/2	3/4	GOF (42, 43)
**rs1469124637**	NA.	58.06	0/7	1/2	NA.	**rs13447339**	p.Ile251fsx	1/1	NA.	NA.	LOF (19)
**rs549486362**	NA.	NA.	0/1	1/1	NA.						
**Del_G**	NA.	NA.	0/1	1/1	NA.						
**Inser_GCGCCT**	NA.	NA.	0/1	1/1	NA.						

^1^AA change: amino acid exchange; ^2^Cper(%): conservation percentile among human gDNA and other 30 species; ^3^Deleteriousness: the deleteriousness of nucleic acid change was evaluated in seven in-silico predictors; ^4^i/n: i = the number of tools which predicted the tested variant as pathogenic based on single nucleotide changing, n = the number of accessible predictors; ^5^Splicing site: the putative changes of tested variants on splicing site of mRNA; ^6^j/k: j = the number of online software which denoted the splicing site may change due to the tested variant, k = the number of accessible software; ^7^Protein stability: the likely changes on protein stability caused by the tested variant based on amino acid sequence or 3D model of protein; ^8^z/g: z = the number of predictors which declared the stability of protein decreased, g = the number of accessible software; ^9^RF: reduced function; ^10^LOF: loss of function; ^11^GOF: gain of function; ^12^Parameters in bold: pathogenic in all available predictors; ^13^NA.: not applicable.

##### 3.2.1.3 One detected intronic variant in strong linkage disequilibrium with one BMI GWAS hit

Two GWAS summary statistic datasets [BMI GWAS by Pulit et al. (29) and AN GWAS by Watson et al. (30)] were used to analyze the putative association between *LCN2* and the two traits. Thus, the plots of the *LCN2* genomic region ± 500kb denoted that no AN and BMI GWAS hit located within *LCN2* and a few variants in the downstream ~ 100kb associated with BMI [(plots are shown in **Supplementary Material Figure 1**)], data extracted from GWAS were collected in **Supplementary Material Tables 1, 2**. The detected variants with dbSNP IDs and those BMI GWAS hits were calculated LD scores in LDmatrix (**Supplementary Material Figure 2**, **Table 10-1, 2**).

One detected frequent intronic SNP rs11794980 and one GWAS hit rs2502728 are in strong linkage disequilibrium (R^2^ = 0.346; D’ = 0.622). LDpair showed the haplotypes of these two SNPs (**Supplementary Material Figure 3**]). BMI GWAS hit rs2502728(T) minor allele is in LD with rs11794980(C) allele (χ^2^ test for haplotypes *p* < 0.0001, distance between two SNPs: ~ 59 kb). The allele T of rs2502728 is associated with increased BMI in the combined sexes GWAS (*p* = 3.28 × 10^-8^, *β* = 0.0097) (29). Other detected variants with known dbSNP IDs were analyzed for haplotype formation with rs11794980 (LDpair tool). All of them are in linkage equilibrium with rs11794980 (**Supplementary Material Tables10-3**.

##### 3.2.1.4 Missense variants causing secondary structure changes in *LCN2*

All five mutated and wildtype amino acid sequences of lipocalin-2 were evaluated in PredictProtein (44). The secondary protein structure is relatively robust and over 50%-80% of point variants may not significantly change the two- and three-dimensional structure of a protein (45). For all five detected point missense variants in *LCN2* the secondary elements were not altered (**Supplementary Material Tables 9-4**). Only the solvent accessibility of polymorphism p.Val89Ile was predicted as ‘exposed’ instead of ‘buried’. Solvent accessibility is essential for determining protein folding patterns and stability in structural bioinformatics (46). Variants p.Leu6Pro and p.Gly9Val are located in the signal peptide region of lipocalin-2. Signal peptides mediate the targeting of precursor secretory proteins to the correct organelle, such as cell membrane or endoplasmic reticulum (47). Besides, it was demonstrated that signal peptides control the secretion of protein by preventing the premature or misfolding of secretory proteins (48).

#### 3.2.2 Analyses for *MC4R* missense variants

Twelve NSVs in *MC4R* were analyzed with ELISA assays. Based on the previous studies on functions of variants, including three frameshift variants, they were classified into GOF (two NSVs), RF (five NSVs) and LOF (five NSVs). Based on the *in-silico* prediction eight of the nine single nucleotide alterations were predicted as pathogenic in all dimensions [(**Table 3**, detailed information in **Supplementary Material Tables 11, 12**)].

### 3.3 ELISA assays for lipocalin-2 serum levels

Circulating lipocalin-2 levels were measured in two heterozygous *LCN2*-variant carriers (p.Val89Ile and p.Asp44Asn), 33 *MC4R*-variant carriers and 33 female matched controls (age/LBM%/diagnosis) without *MC4R* or *LCN2* variants.

#### 3.3.1 Negative correlation between BMI and LBM%

**Figure 3:**
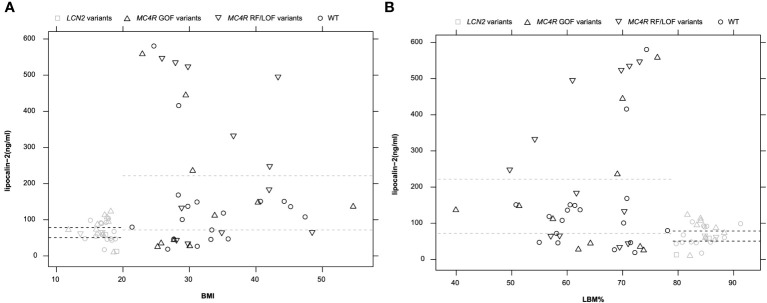
Circulating lipocalin-2 level in 68 analyzed individuals including *LCN2*-variant carriers (n = 2), *MC4R*-variant carriers (n = 33), and individuals without variant in these two genes (n = 33). **(A)**: The pattern of lipocalin-2 levels of all samples was shown along with BMI. **(B)**: LBM% as a scaled parameter for lipocalin-2 levels (one sample, p.Val103Ile_k, was excluded from this figure due to missing LBM% value). Dashed lines indicate the 95% CI of lipocalin-2 in serum in groups of individuals with leanness or obesity. Black symbols indicate obese individuals and grey symbols indicate individuals with leanness.

All 68 analyzed individuals were plotted in **Figure 3** with lipocalin-2 levels and BMI or LBM%. The analyzed group was firstly divided by diagnosis, no difference could be observed between healthy-lean individuals and the females with acute or recovered AN (**Supplementary Material Figure 4**, **Supplementary Material Tables 13-2**). The analyzed group was then divided into lean (both healthy lean individuals and females with AN were included) and obese groups (the patients with obesity). There was no overlap between the lipocalin-2 serum concentrations between the two groups. The opposite pattern of lipocalin-2 scaled by BMI and LBM% were shown. BMI was negatively correlated with LBM% (Spearman’s rho = -0.975, *p* < 0.001), whereas BMI was positively correlated with LBM (Spearman’s rho = 0.866, *p* < 0.001) based on 33 individuals without variants in the *MC4R* and *LCN2* genes. Thus, the LBM of patients with obesity increased with BMI, while the percentage of LBM decreased (**Supplementary Material Tables 13-1**). However, no correlation between BMI or LBM% and lipocalin-2 serum levels could be observed (**Supplementary Material Tables 13-1**).

#### 3.3.2 Low lipocalin-2 level in the *LCN2*-p.Val89Ile carrier

The genotypes and phenotypes of included individuals are shown in the **Supplementary Materials Tables 4-1**. In two individuals who carried *LCN2* variants (p.Asp44Asn heterozygote in a female with acute AN and p.Val89Ile heterozygote in a female recovered from AN) lipocalin-2 levels were analyzed. We detected a low concentration of serum lipocalin-2 in the p.Val89Ile heterozygous carrier. This female had a lower lipocalin-2 level (average value: 12.5 ng/ml) than the lower bound of the 95% CI for lean individuals (50.47 ng/ml), and its matched controls (average value: 57.36 ng/ml) (**Supplementary Material Tables 4-1-3**).

#### 3.3.3 No significant effect of *MC4R* missense variants on lipocalin-2 levels

The *MC4R* variants with functional classifications were divided into three groups (GOF: gain of function, RF: reduced function, LOF: loss of function) and concentration of lipocalin-2 in serum (ng/ml) within each group was described (**Supplementary Material Tables 4-1**). The two non-synonymous *MC4R* polymorphisms (p.Val103Ile and p.Ile251Leu) lead to a gain of function (19, 35, 36, 42, 43). Detected LOF *MC4R* mutations in our previous studies consisted of two non-synonymous variants [p.Ser127Leu (38), p.Arg165Trp (39, 40)] and three frameshift mutations [p.Try35stop (33, 34), p.Leu211fsX (41), p.Ile251fsX (19)]. Besides, five non-synonymous variants [p.Ser30Phe (33), p.Val50Leu (33), p.Thr112Met (14, 37), p.Ile121Thr (19, 35), p.Ala175Thr (34)] with reduced MC4R protein function were included. The *MC4R* variants which lead to reduced function were considered together (RF and LOF) in **Figure 3**. However, there is no significant difference between RF/LOF and GOF.

#### 3.3.4 Positive correlation between BMI and lipocalin-2 levels

**Figure 3** showed a clear difference between patients with obesity and those with low BMI (healthy lean and patients with AN). The independent samples Mann-Whitney U tests were applied in subgroups to reveal the impact of *MC4R* variants or body shape on lipocalin-2 levels. To exclude the effects of variants, the test was first examined in individuals with normal *LCN2* and *MC4R* genotypes (**Supplementary Material Tables 13-3**). Increased lipocalin-2 levels (*p* = 0.033) could be observed between individuals with obesity (n = 18) compared to lean individuals (n = 15). When the effects of *MC4R* variants were considered, BMI in the individuals with *MC4R* variants between lean (n = 11) and individuals with obesity (n = 22) was not significant (*p* = 0.105) (**Supplementary Material Tables 13-4**).

### 3.4 Additional genotyping for the *LCN2*-p.Val89Ile variant

The circulating lipocalin-2 level of the patient recovered from AN who carried the rare *LCN2* NSV p.Val89Ile heterozygously (12.5 ng/ml) was reduced compared to the lower bound of 95% CI of lean individuals (lower bound: 50.47 ng/ml), the mean value of two matched controls (57.36 ng/ml), and one female with acute AN who carried another *LCN2* missense variant (p.Asn44Asp, 58.45 ng/ml). Thus, a TaqMan assay ensued for p.Val89Ile in an additional study group (170 female patients with AN [acute and recovered] and 200 children or adolescents with severe obesity). We detected a female patient with severe obesity also heterozygous for p.Val89Ile. However, a serum sample of this patient was not available (**Table 2**).

## 4 Discussion

Numerous studies showed elevated lipocalin-2 levels in patients with obesity (11, 49, 50). Lipocalin-2 is highly expressed by fat cells both *in-vivo* and *in-vitro* (10). Mosialou et al. described that lipocalin-2 regulates body weight by binding to the MC4R in the hypothalamus (11). In our study, Sanger sequencing for the *LCN2* gene, *in-silico* analyses for detected variants, and ELISA assays for *LCN2* or *MC4R* variants carriers and matched controls were performed.

### 4.1 *LCN2* is associated with both body weight regulation and AN

By Sanger sequencing, 14 variants were detected in *LCN2*. Two missense variants (p.Leu6Pro and p.Gly9Val) were highly conserved. predicted as pathogenic in all dimensions of *in-silico* tools and located in the signal peptide structure of lipocalin-2. Thus, they are highly likely to induce functional consequences for protein structure and function. A detected frequent intronic SNP rs11794980 strongly linked to one BMI GWAS hit rs2502728 (R^2^ = 0.346, D’ = 0.622) (29). Allele C of rs11794980 is likely to be inherited with the minor allele T of rs2502728 which is associated with increased BMI (combined sexes BMI: *β* = 0.0097, *p* = 3.3 x 10^-8^) (29). Thus, the infrequent C allele rs11794980 may be associated with increase body weight.

### 4.2 Non-synonymous variant p.Val89Ile in *LCN2* may decrease circulating lipocalin-2 level

Due to the serum sample limitation, only for two of five non-synonymous *LCN2* variants (p.Asp44Asn and p.Val89Ile) ELISA assays were used to evaluate circulating lipocalin-2 levels in heterozygous carriers. Lipocalin-2 is mainly secreted by osteoblasts and the ratio of adipocytes to bone cells can be reflected by adipose marrow (22). MRI assessments showed that the adipose marrow increased in patients with acute AN (51, 52), and no difference was observed between patients with recovered AN and healthy individuals (53). Moreover, the two variants were predicted as pathogenic in all three dimensions of computational annotations, whereas p.Asp44Asn is in a higher conservation position (80.65%) than p.Val89Ile (16.13%). Thus, we expected p.Asp44Asn may decrease the stability of lipocalin-2 and downregulate lipocalin-2 levels. However, lower serum level of lipocalin-2 was detected in the heterozygote of p.Val89Ile.

*LCN2*-p.Val89Ile may change the solvent accessibility of protein from ‘buried’ to ‘exposed’. Around 67% of wild type amino acid residues related to diseases were located in the buried position of protein (54), which may imply that the solvent accessibility of the 89^th^ amino acid of lipocalin-2 has an impact on protein structure and might be associated with diseases. Moreover, the hydrophobic scale of isoleucine is higher than valine depending on all five popular calculation methods (55–59). ELISA assays of the p.Val89Ile NSV carrier (one female recovered from AN) showed a lower lipocalin-2 level in serum than all other comparable groups (lower bound of 95% CI, matched controls, another *LCN2* NSV carrier). The low expression level of lipocalin-2 may be due to the changed solvent accessibility and decreased protein stability.

### 4.3 Multiple factors can influence lipocalin-2 levels in wildtype *LCN2* individuals

Lipocalin-2 is secreted by hematopoietic and BMA cell types. The bone mass of young premenopausal women is significantly correlated to LBM (22–24, 60). Thus, when bone mass cannot be measured, LBM can be used as an indicator of presumed bone mass and the amount of lipocalin-2 secretion. LBM is influenced directly by body weight. Thus we used normalized LBM (LBM%, normalized by body weight). LBM% and BMI are significantly negatively correlated. Here, we analyzed LBM% and BMI in all individuals screened for lipocalin-2 level. However, the calculation of LBM in our study is based on formulas and might lead to inaccurate or ambiguous results. In future investigations, an actual bone mass and body composition could be determined by MRI and may show a more defined correlation between body composition and lipocalin-2 levels.

#### 4.3.1 High BMI is a factor that can influence lipocalin-2 serum levels

A previous study reported that lipocalin-2 levels were positively correlated to BMI (50). We hypothesized significantly different lipocalin-2 levels existed in individuals with low BMI (healthy lean individuals and females with AN) and patients with obesity. Mean serum lipocalin-2 levels were increased in obese versus lean individuals (*p* = 0.033). Thus, lipocalin-2 levels in individuals (normal genotypes at *LCN2* and *MC4R*, excluded the impact of NSVs) with a BMI above 30 kg/m^2^ or BMI percentile greater than 97^th^ are likely to have a higher lipocalin-2 concentration in serum than controls with normal body weight. In future investigations, it might be possible to use MRI or dual-energy X-ray absorptiometry to determine the actual bone mass of individuals, which may show a clearer pattern between body composition and lipocalin-2 levels. Previous studies reported that the putatively negative correlation between BMA percentile and lipocalin-2 levels (22, 61) and the increased BMA percentile in patients with acute AN and severe obesity (22, 52, 62), determination of the bone components may reveal the impact of diseases on lipocalin-2 secretion.

#### 4.3.2 *MC4R* variants may influence lipocalin-2 levels

Although tests for the impact of *MC4R* variants were not significant in both groups (lean or obese individuals), the non-significance might be due to the multi-direction of *MC4R* variants on the protein function (GOF, RF, or LOF). The small sample size reduces the power of our analysis and limits the analysis methods that can be used. Thus, it is impossible to analyze the impact of *MC4R* variants according to variants catalogs or single variants.

Many genes are involved in the leptin-melanocortin pathway that has been associated with monogenic obesity through their influence on food intake and energy expenditure (63). Thus, the varied expression of MC4R may affect lipocalin-2 expression and the increasing BMI or decreasing LBM% can elevate lipocalin-2 in serum secretion. However, the mutations of diminished function may be compensated by other factors so that the potential pattern of lipocalin-2 secretion is not observed. Thus, we cannot confirm that a feedback loop exists between lipocalin-2 and MC4R, but the results of ELISA implied that protein function changes of MC4R might influence lipocalin-2 concentration in serum.

Limitations of our study include the relatively low number of mutation carriers that could be used for the lipocalin level analyses and the lack of functional *in-vitro* studies.

## 5 Conclusion

We detected fourteen variants in *LCN2*, including five non-synonymous variants. The highly conserved variants p.Leu6Pro and p.Gly9Val are located in the signal peptide region of the lipocalin-2 and might result in functional consequences. According to the GWAS datasets, LD analyses, and gene network look-up, *LCN2* might be relevant for both AN and body weight regulation. A low lipocalin-2 level in the female who carried *LCN2* NSVp.Val89Ile and recovered from AN was observed. This might be caused by decreased protein stability, the increased hydrophobic scale of the protein and altered solvent accessibility. The ELISA assays of all *MC4R* and *LCN2* wildtype samples implied increased lipocalin-2 levels in the individuals with high BMI (*p* = 0.033). Although no clear pattern of impacts of single *MC4R* variants on lipocalin-2 levels was found, an additional ELISA assay with an expanding sample size of variant carriers may reveal the masked pathway between *LCN2* and *MC4R*.

## Data availability statement

The original contributions presented in the study are included in the article/**Supplementary Material**. Further inquiries can be directed to the corresponding author.

## Ethics statement

Written informed consent was given by all underage participants and in case of minors by their parents. The study was approved by the Ethics committees of the Universities of Essen, Marburg, Aachen, Dresden, Frankfurt, Hannover, Heidelberg, Tübingen and Würzburg and was performed in accordance with the Declaration of Helsinki.

## Author contributions

YZ and AH designed the study. DF, ST, BH-D, JS, MdZ, WH, SE, SZ, KG, KE, RB, and MF recruited the probands. YZ and AH were responsible with the experimental design. YZ and JG performed the molecular genetic experiments and assembled the datasets. DZ and MK performed the ELISA analysis. YZ performed the bioinformatic analyses. YZ and TP performed the statistical analyses. YZ, LR, JH, SA-L and AH interpreted the data. YZ wrote the draft of the manuscript and included input of all authors. All authors contributed to the article and approved the submitted version.
