# Supplementary material for: Lipocalin 2 – mutation screen and serum levels in patients with anorexia nervosa or obesity and in lean individuals
Source: Front Endocrinol (Lausanne). 2023 Mar 21;14:1137308. doi: 10.3389/fendo.2023.1137308 (PMC10071025; doi:10.3389/fendo.2023.1137308)
Supplement: Supplementary file 1 [file Image_1.pdf]

Supplementary Material Figures

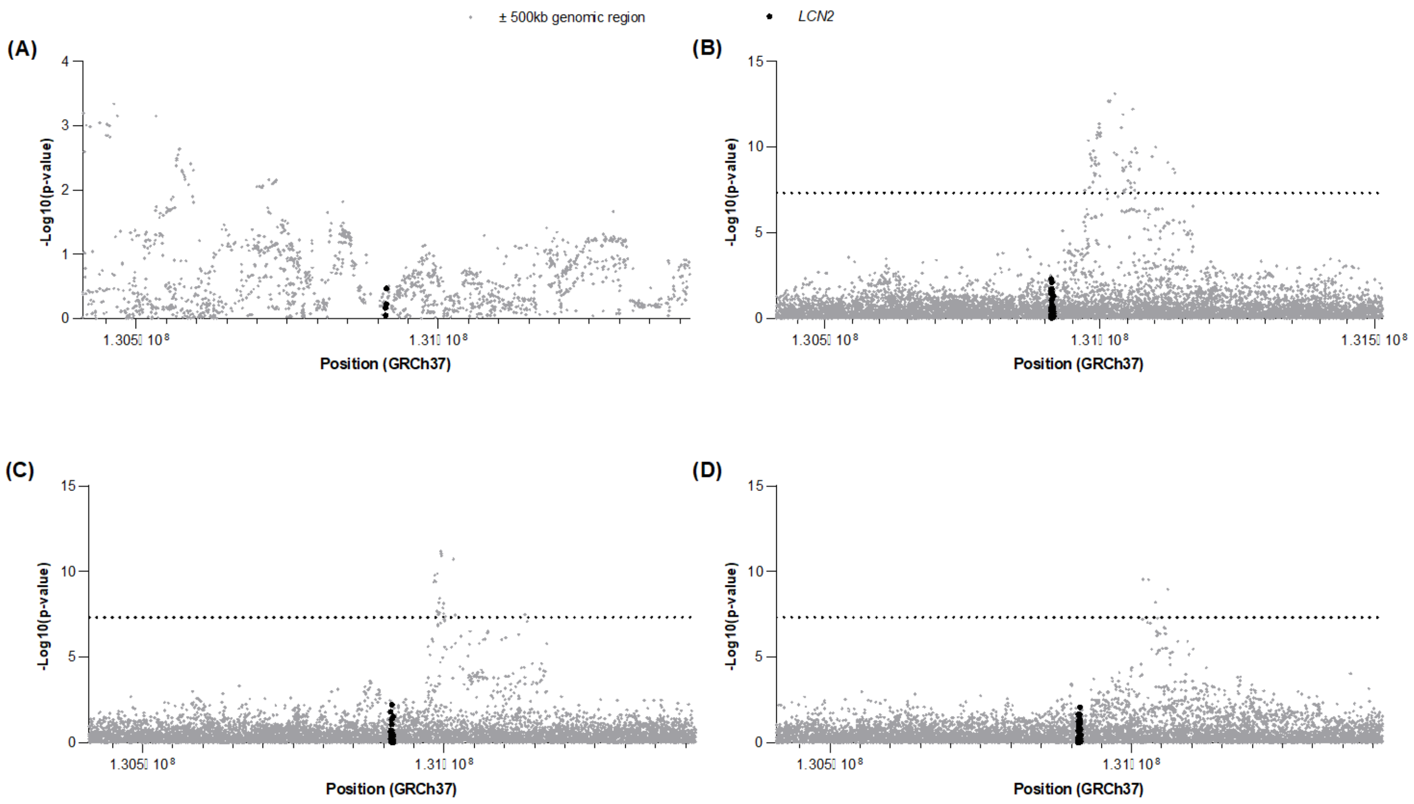

Figure 1. Plots of the chromosomal location and p values of variants located near *LCN2* (± 500 kb) on BMI GWAS and AN GWAS data. Variants were extracted from Pulit et al. BMI GWAS (1) and Watson et al. AN GWAS (2). **(A)**: AN, **(B)**: BMI (combined sexes), **(C)**: BMI males, **(D)**: BMI females.

The −log10(p value) for the association are shown on the y-axis and the chromosomal locations are ordered on the x-axis. The variants which surpassed the dashed line are genome-wide significant. Light gray dots indicate ± 500 kb genomic region of *LCN2*, variants located in the *LCN2* genomic region plotted with black dots.

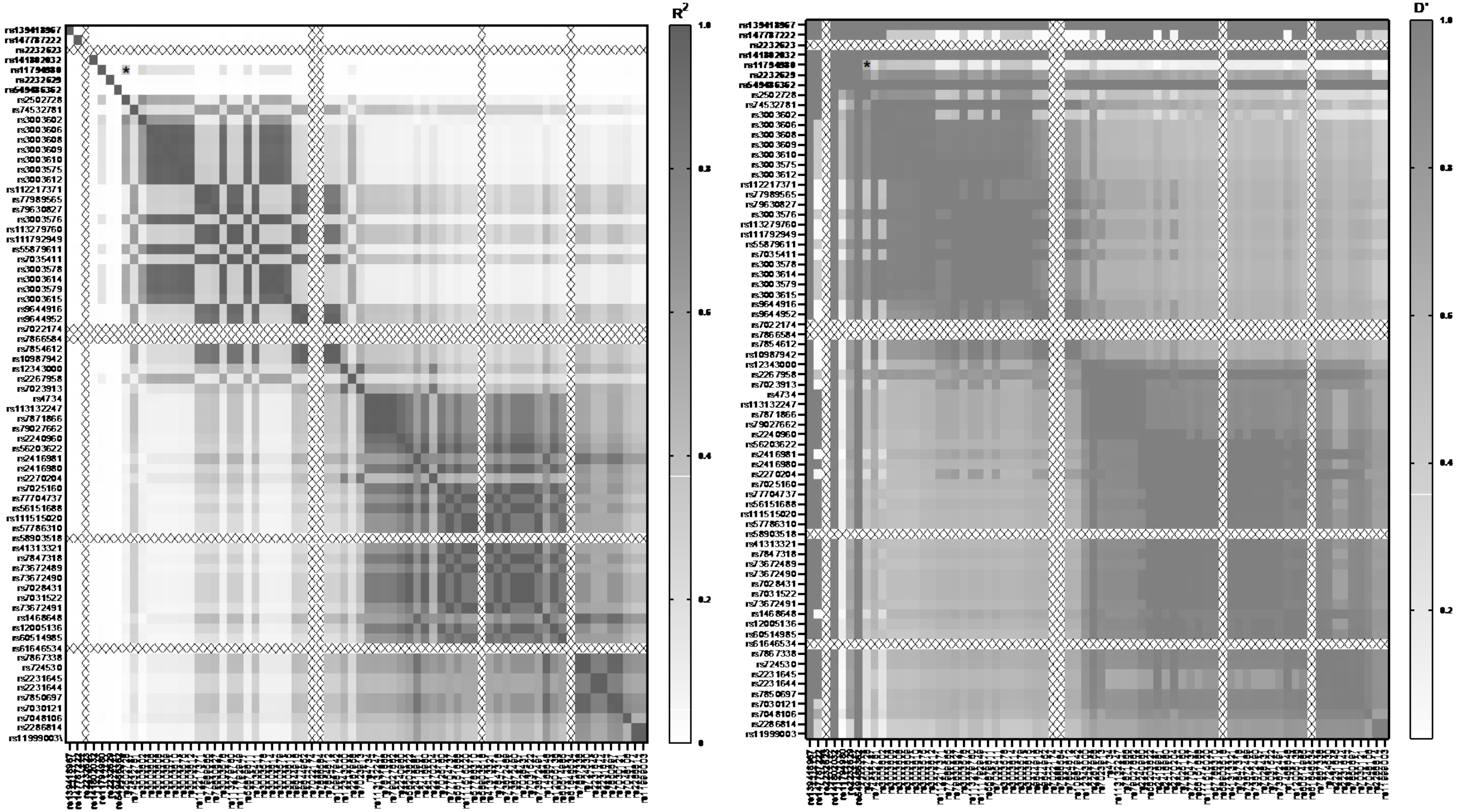

Figure 2. LD matrix of detected variants in *LCN2* and genome wide significant SNPs extracted from Pulit et al. BMI combined sexes GWAS summary statistic dataset (1). Detected variants in *LCN2* were in bold; * indicated the pair of SNPs in strong LD (D' > 0.6, R^2 > 0.3).

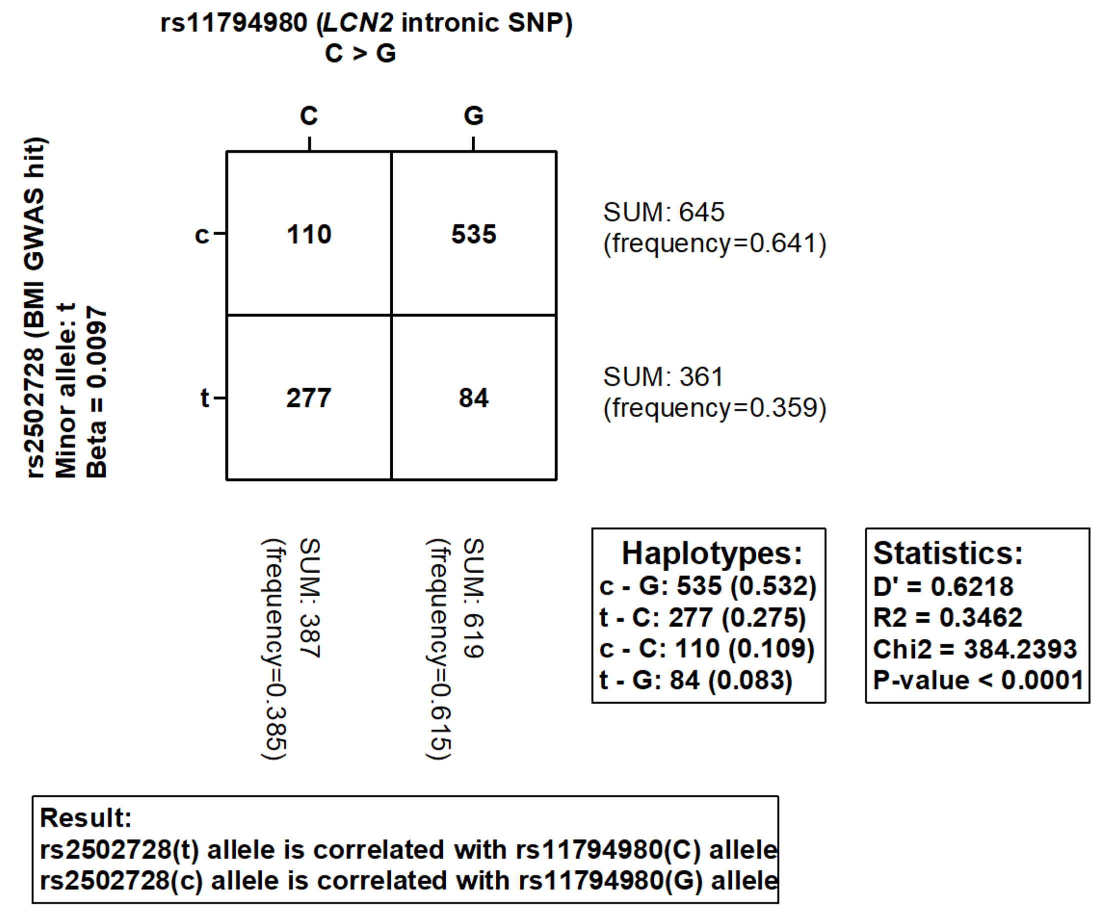

Figure 3. Haplotype of detected intronic *LCN2* SNP rs11794980 and BMI GWAS (Pulit et al. BMI GWAS combined sexes) hit rs2502728 (1).

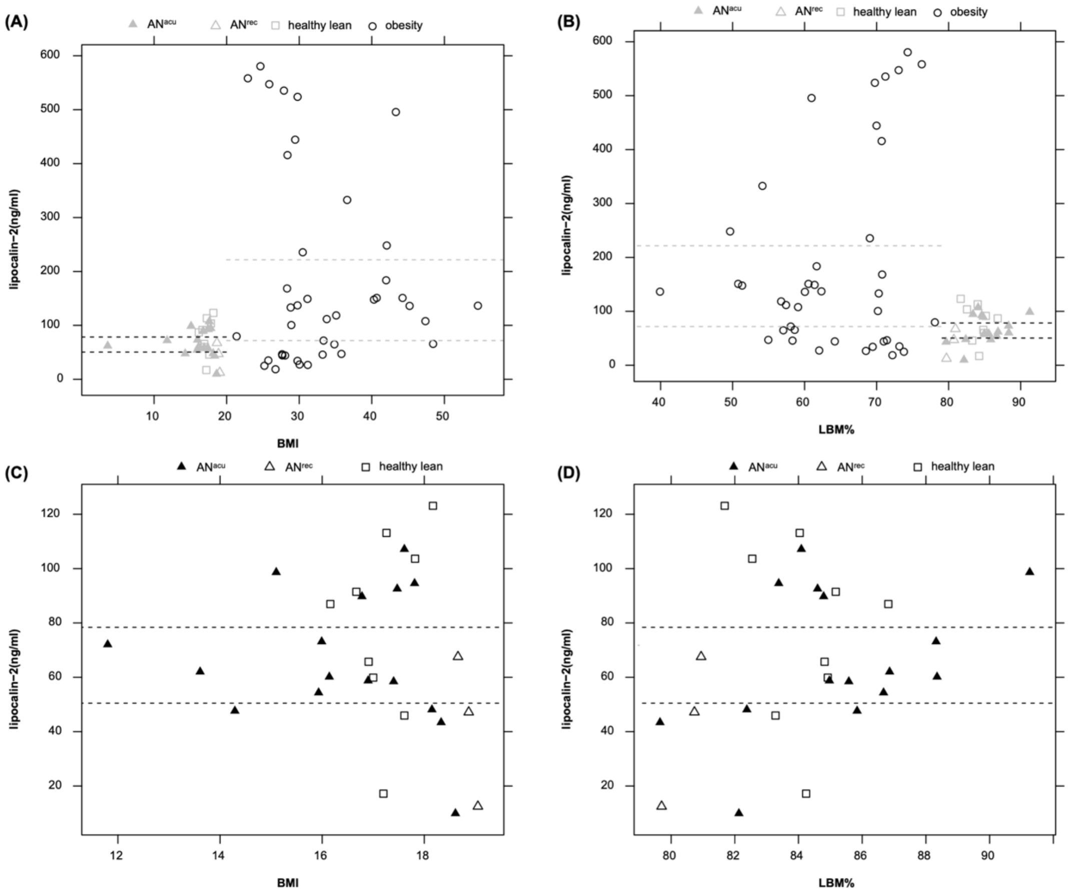

Figure 4 Circulating lipocalin-2 level in 68 analyzed individuals including *LCN2*-variant carriers (n = 2), *MC4R*-variant carriers (n = 33), and individuals with normal genotypes of *LCN2* and *MC4R* (n = 33). (A): The pattern of lipocalin-2 levels of all samples was shown along with BMI. **(B)**: LBM% as a scaled parameter for lipocalin-2 levels of 67 samples (one sample, p.Val103Ile_k, was excluded from this figure due to the missing of LBM% value). **(C)**: The pattern of lipocalin-2 levels of all lean individuals (patients with AN and healthy lean) was shown along with BMI. **(D)**: LBM% as a scaled parameter for lipocalin-2 levels of lean individuals (patients with AN and healthy lean, one sample, p.Val103Ile_k, was excluded from this figure due to the missing of LBM% value). Dashed lines indicated the 95% CI of lipocalin-2 in serum for lean and obese control groups.

Reference:

1. Pulit SL, Stoneman C, Morris AP, Wood AR, Glastonbury CA, Tyrrell J, et al. Meta-analysis of genome-wide association studies for body fat distribution in 694 649 individuals of European ancestry. Human Molecular Genetics. 2018;28(1):166-74.

2. Watson HJ, Yilmaz Z, Thornton LM, Hübel C, Coleman JR, Gaspar HA, et al. Genome-wide association study identifies eight risk loci and implicates metabo-psychiatric origins for anorexia nervosa. Nature genetics. 2019;51(8):1207-14.
