# Supplementary material for: Lipocalin 2 – mutation screen and serum levels in patients with anorexia nervosa or obesity and in lean individuals
Source: Front Endocrinol (Lausanne). 2023 Mar 21;14:1137308. doi: 10.3389/fendo.2023.1137308 (PMC10071025; doi:10.3389/fendo.2023.1137308)
Supplement: Supplementary file 3 [file DataSheet_2.docx]

**Supplementary Materials Methods**

**M1 LBM calculation**

James’ formula is not appropriate for individuals with high BMI. The LBM calculated by Boer and Hume formulas, on the other hand, is even higher than the total body weight in some individuals with severely low BMI (1). Therefore, James formula was applied only in the underweight adolescent or adult individuals (BMI < 15 kg/m^2^) (2). The average values of LBM calculated with Boer’s and Hume’s formulas were applied for adolescents with obesity(3, 4). The mean values of results generated form all three formulas were applied for healthy-lean individuals and patients with AN (BMI ≥ 15 kg/m^2^). Peters’ formula, suitable for the children (5), was used to calculate the LBM for all individuals younger than 14 years.

**The Boer Formula** (3):

$${LBM}_{female}=0.252\times Weight\left( kg \right)+0.473\times Height\left( cm \right)-48.3$$

$${LBM}_{male}=0.407\times Weight\left( kg \right)+0.267\times Height\left( cm \right)-19.2$$

**The James Formula** (6):

$${LBM}_{female}=1.07\times Weight\left( kg \right)+148\times\left( \frac{Weight\left( kg \right)}{Height\left( cm \right)} \right)^{2}$$

$${LBM}_{male}=1.1\times Weight\left( kg \right)+128\times\left( \frac{Weight\left( kg \right)}{Height\left( cm \right)} \right)^{2}$$

**The Hume Formula** (4):

$${LBM}_{female}=0.29569\times Weight\left( kg \right)+0.41813\times Height\left( cm \right)-43.2933$$

$${LBM}_{male}=0.3281\times Weight\left( kg \right)+0.33929\times Height\left( cm \right)-29.5336$$

**The Peters Formula** for children (younger than 14 years old) (5):

$${LBM}_{child}=0.0817\times{Weight(kg)}^{0.6469}\times{Height(cm)}^{0.7236}$$

**M2 Primer pairs for amplifying *LCN2* coding region**

| Primer name | Primer Sequence | Amplified Exon | Fragment length (bp) | Chromosome range (GRCH38) |
| --- | --- | --- | --- | --- |
| *LCN2*_E2F* | AAGAATGAATCAACCCACCC | Exon 2 | 401 | 9: 128,149,396 ~ 128,149,796 |
| *LCN2*_E2R | GAGTGAGCTGAAACCTGGAC |  |  |  |
| *LCN2*_E3F | CACAGGGACAGAGCTGGAG | Exon 3 | 402 | 9: 128,150,167 ~ 128,150,568 |
| *LCN2*_E3R | GTCCATCCCCACCACTGT |  |  |  |
| *LCN2*_E4~6F | TGTCAGCTCAGGGAGAAGG | Exon 4 ~ Exon 6 | 972 | 9: 128,151,438 ~ 128,152,409 |
| *LCN2*_E4~6R | GACCATCCACTGCTCCAG |  |  |  |
| *LCN2*_E7F | CTGCCTGGACTGGTAAACA | Exon 7 | 497 | 9: 128,152,807 ~ 128,153,303 |
| *LCN2*_E7R | GAGGTGGATATGAGGGTGAT |  |  |  |

*The first Exon of *LCN2* does not translate amino acid, thus we didn’t sequence this exon.

**M3 *In-silico* analyses for detected *LCN2* and analyzed *MC4R* variants via 12 softwares**

MutationTaster (https://www.mutationtaster.org/) was utilized to estimate variants’ potential deleteriousness including deletions and insertions (7). Then the effects of point mutations were evaluated in Combined Annotation dependent depletion (CADD, https://cadd.gs.washington.edu/snv) (8), PredicSNP2 (https://loschmidt.chemi.muni.cz/predictsnp2/ ) (9) that evaluates the value of DANN, FATHMM, FunSeq2 and GWAVA as well. The base pair exchange may affect the mRNA structure or stability or the splicing regulation. Thus, all variants were analyzed in ESEfinder 3.0 (http://rulai.cshl.edu/cgi-bin/tools/ESE3/esefinder.cgi?Process=home) (10) and Spliceman (http://fairbrother.biomed.brown.edu/spliceman/ ) (11) to quantitative the putative changing in mRNA splicing pattern.

For the missense variants, which alter the amino acid, after the deleteriousness analyses in PolyPhen2.0 (http://genetics.bwh.harvard.edu/pph2/) (12) and PROVEAN (http://provean.jcvi.org/index.php ) (13), the protein sequence and model were used to analyze if the mutation alters the structure or stability of the mature protein. The 3D model of LCN2 and MC4R were evaluated in I-Mutant3.0 (http://gpcr2.biocomp.unibo.it/cgi/predictors/I-Mutant3.0/I-Mutant3.0.cgi) (14) and SDM (<http://marid.bioc.cam.ac.uk/sdm2>) (15) (PDB-id: 3U0D and 7AUE, respectively for LCN2 and MC4R). The other missense variants which are not available in 3D models were analyzed with their amino acid sequence in I-Mutant2.0 (https://folding.biofold.org/i-mutant/i-mutant2.0.html) (16) and MUpro (http://mupro.proteomics.ics.uci.edu/) (17). An online software PredictProtein (https://predictprotein.org/) can predict the possible secondary structure of protein based on the amino acid sequence and simulate the protein secondary structure alteration due to amino acid exchanges (18). Five mutated LCN2 amino acid sequences with detected variants have been compared to the wildtype amino acid sequence (extracted from Ensembl GRCh 38.p13). In this software, secondary structure elements, such as helix, stand or other structures, and the solvent accessibility of protein molecules were predicted.

**M4 Statistical analyses for detected *LCN2* variants**

Statistic for detected variants in *LCN2* was performed in two sections: (1) detected variants via Sanger Sequencing, (2) three missense variants (p.Gly9Val, rs147787222; p.Val89Ile, rs200876706; p.Arg174Ser, rs546790138) via Sanger Sequencing and TaqMan assays. The Hardy-Weinberg Equilibrium analysis (HWE) was performed to check data quality and model assumptions.

Association between alleles/genotypes and disease was described with Fisher’s exact test(https://www.socscistatistics.com/tests/fisher/default2.aspx). For the variants that were detected in cases (AN, obesity), the genotypes extracted from Gnomad V3.1.1 non-Finnish European (<https://gnomad.broadinstitute.org/>) were applied as controls. The rare variants, which cannot find genotypes or detected once in Gnomad, were excluded from our study. For multiple tests, the significant thresholds were adjusted with Bonferroni correction (adjusted *p* = 0.05/*n*, where *n* is the number of multiple tests).

**Reference:**

1. Caruso D, De Santis D, Rivosecchi F, Zerunian M, Panvini N, Montesano M, et al. Lean body weight-tailored iodinated contrast injection in obese patient: Boer versus James formula. BioMed Research International. 2018;2018.

2. Organization WH, Organization WH. The international classification of adult underweight, overweight and obesity according to BMI. Geneve: World Health Organization. 2004.

3. Boer P. Estimated lean body mass as an index for normalization of body fluid volumes in humans. American Journal of Physiology-Renal Physiology. 1984;247(4):F632-F6.

4. Hume R. Prediction of lean body mass from height and weight. Journal of clinical pathology. 1966;19(4):389-91.

5. Peters A, Snelling H, Glass D, Bird N. Estimation of lean body mass in children. British journal of anaesthesia. 2011;106(5):719-23.

6. Research DMGoO, James WPT, Waterlow JC. Research on Obesity: a Report of the DHSS/MRC Group; Compiled by WPT James: HM Stationery Office; 1976.

7. Schwarz JM, Cooper DN, Schuelke M, Seelow D. MutationTaster2: mutation prediction for the deep-sequencing age. Nature methods. 2014;11(4):361-2.

8. Rentzsch P, Witten D, Cooper GM, Shendure J, Kircher M. CADD: predicting the deleteriousness of variants throughout the human genome. Nucleic acids research. 2019;47(D1):D886-D94.

9. Bendl J, Musil M, Štourač J, Zendulka J, Damborský J, Brezovský J. PredictSNP2: a unified platform for accurately evaluating SNP effects by exploiting the different characteristics of variants in distinct genomic regions. PLoS computational biology. 2016;12(5):e1004962.

10. Cartegni L, Wang J, Zhu Z, Zhang MQ, Krainer AR. ESEfinder: a web resource to identify exonic splicing enhancers. Nucleic acids research. 2003;31(13):3568-71.

11. Lim KH, Fairbrother WG. Spliceman—a computational web server that predicts sequence variations in pre-mRNA splicing. Bioinformatics. 2012;28(7):1031-2.

12. Adzhubei I, Jordan DM, Sunyaev SR. Predicting functional effect of human missense mutations using PolyPhen‐2. Current protocols in human genetics. 2013;76(1):7.20. 1-7.. 41.

13. Choi Y, Sims GE, Murphy S, Miller JR, Chan AP. Predicting the functional effect of amino acid substitutions and indels. 2012.

14. Capriotti E, Calabrese R, Casadio R. Predicting the insurgence of human genetic diseases associated to single point protein mutations with support vector machines and evolutionary information. Bioinformatics. 2006;22(22):2729-34.

15. Pandurangan AP, Ochoa-Montaño B, Ascher DB, Blundell TL. SDM: a server for predicting effects of mutations on protein stability. Nucleic acids research. 2017;45(W1):W229-W35.

16. Capriotti E, Fariselli P, Casadio R. I-Mutant2. 0: predicting stability changes upon mutation from the protein sequence or structure. Nucleic acids research. 2005;33(suppl_2):W306-W10.

17. Cheng J, Randall A, Baldi P. Prediction of protein stability changes for single‐site mutations using support vector machines. Proteins: Structure, Function, and Bioinformatics. 2006;62(4):1125-32.

18. Bernhofer M, Dallago C, Karl T, Satagopam V, Heinzinger M, Littmann M, et al. PredictProtein - Predicting Protein Structure and Function for 29 Years. Nucleic Acids Research. 2021;49(W1):W535-W40.
